# The impact of alternate nostril breathing on the severity and frequency of migraine attacks: a randomized control trial

**DOI:** 10.1017/S1463423625000064

**Published:** 2025-02-14

**Authors:** Oğulcan Çöme, Gizim Limnili, Azize Dilek Güldal

**Affiliations:** Department of Family Medicine, Dokuz Eylül University Faculty of Medicine, Izmir, Turkey

**Keywords:** Breathing techniques, Migraine, migraine disability, non-pharmacological therapy, randomized controlled trial

## Abstract

**Background:**

Migraine is a prevalent and debilitating neurological disorder that significantly affects quality of life. While pharmacological treatments exist, they can have limitations such as side effects, contraindications, and incomplete relief, prompting interest in non-pharmacological approaches for better symptom management.

**Objective:**

This study aimed to assess the effectiveness of alternate nostril breathing (ANB) as a non-pharmacological intervention to reduce the frequency and severity of migraine attacks and associated disability in adult patients.

**Methods:**

A single-center, open-label, two-arm, parallel-group randomized controlled trial was conducted at six Family Health Centers (FHCs) of Dokuz Eylul University, Izmir, Turkey. A total of 86 migraine patients aged 18–50 years, diagnosed with migraine based on ICD-10 criteria, were randomized into control (n = 43) and intervention (n = 43) groups. The intervention group practiced ANB three times daily for three months, while the control group continued their usual care. The primary outcomes were changes in migraine frequency and severity. Secondary outcomes included changes in migraine-related disability, both outcomes measured using the Migraine Disability Assessment Scale (MIDAS).

**Results:**

The intervention group showed a significant reduction in migraine attack frequency (*P* = 0.002) and MIDAS scores (*P* = 0.003) compared to the control group. Both groups experienced a reduction in attack severity (*P* = 0.001), though no significant difference was observed between the groups (*P* = 0.074). Within-group comparisons showed significant improvements in attack frequency, severity, and MIDAS scores in the intervention group (*P* = 0.001 for all).

**Conclusion:**

ANB significantly reduced migraine frequency and disability, making it a promising non-invasive and accessible treatment option for migraine management. Further research with longer follow-up periods is needed to explore its long-term effects and broader applicability.

## Introduction

Migraine is a prevalent neurobiological disorder characterized by increased central nervous system excitability. Diagnosing migraine involves identifying specific headache characteristics and accompanying symptoms. The primary symptom is a pulsating, severe, typically unilateral headache, often accompanied by increased sensitivity to light and sound, nausea, and vomiting. Migraine attacks, lasting 4 to 72 h, are frequently preceded by visual, auditory, and sensory auras (Amiri *et al.*, 2022).

Migraine is one of the most common neurological disorders worldwide. The global prevalence of migraine is estimated to be around 14.7%, affecting approximately one billion people, and it is more common in women than men, with a prevalence rate of 18% and 6% in orderly, likely due to hormonal differences (Gupta & Gaurkar, 2022). Chronic migraine affects about 1–2% of the world’s population, with 2.5% of episodic migraine cases progressing to chronic migraine, increasing disability and healthcare utilization (Gawde *et al.*, 2023).

Pharmacological treatments for migraine include a variety of medications designed to alleviate symptoms and reduce the frequency of attacks. Acute treatments aim to relieve pain and include nonsteroidal anti-inflammatory drugs, triptans, and ergotamines. Prophylactic treatments are used to prevent migraines and include beta-blockers, antidepressants (Puledda *et al.*, 2023). Despite their effectiveness, these pharmacological therapies have limitations, including side effects, contraindications, and the potential for medication overuse headache. Some patients may also experience inadequate relief or develop tolerance to medications over time, leading to a need for alternative treatment strategies.

Since 1998, researchers have investigated hyperbaric oxygen therapy (HBOT) for treating migraines (Wilson *et al.*, 1998). In their 2021 systematic review, Ciarambino et al. reported evidence suggesting that HBOT may help relieve migraine headaches (Ciarambino *et al.*, 2021). Various studies also indicated that high-flow oxygen therapy (HFOT) for migraine is a safe, effective acute treatment option and may be better tolerated than other treatments (Singhal *et al.*, 2017; Schindler *et al.*, 2018).

However, there are significant limitations to the accessibility and feasibility of HBOT and HFOT for migraine treatment. HBOT requires specialized equipment and facilities, making it inaccessible for many patient. HFOT, while more accessible than HBOT, still requires medical-grade oxygen and proper administration, which may not be feasible for all patients. These limitations highlight the need for more accessible and affordable treatment options for migraine management (Wang *et al.*, 2023).

Breathing techniques in meditation and yoga have been found to have effects comparable to those of HBOT. A 2017 study found that these techniques can increase arterial oxygenation and activate the parasympathetic system by improving ventilation efficiency (Russo *et al.*, 2017). A 2011 study showed that yoga-based respiratory training could lower breathing rates and increase resting oxygen saturation (Santaella *et al.*, 2011). Additionally, a 2021 study indicated that slower breathing rates during yoga might reduce sensitivity to hypoxic and hypercapnic reflexes (Santaella *et al.*, 2011).

A non-invasive technique is alternate nostril breathing (ANB), which improves respiratory function, regulates sympathetic-vagal balance, reduces stress, enhances metabolism, boosts cognitive functions, and mitigates physiological aging (Nivethitha *et al.*, 2016). Regular yoga practice, including ANB, is an excellent exercise for maintaining health across all age groups, beneficial in preventing, controlling, and rehabilitating many diseases.

This study aims to investigate the impact of the ANB technique on the frequency and severity of migraine-type headaches, as well as its effect on migraine-related disability.

## Method

### Study design

This study is designed as a single-center, open-label, two-arm, parallel-group randomized controlled trial (RCT) conducted at six Family Health Centers (FHCs) of Dokuz Eylul University in different regions of Izmir. This study was reviewed and approved by the Ethics Committee of Izmir Dokuz Eylul University, with the file number 7201-GOA. The trial was registered on 09/09/2022 with the registration number ‘NCT05536635’ in the US NIH clinical trials database.

**Figure 1.**
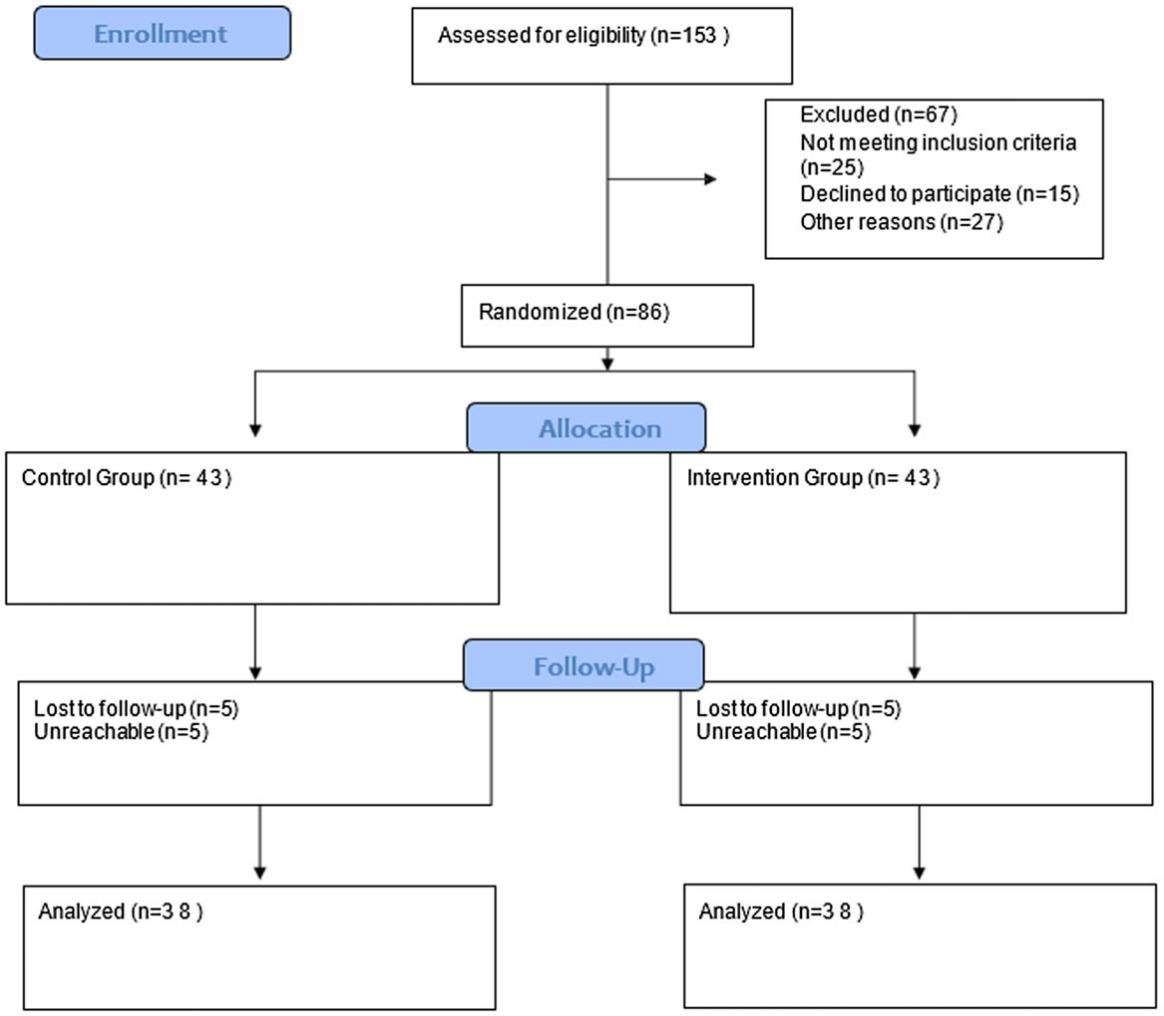
CONSORT diagram.

### Population and sample

The study population comprises patients registered at the FHC of Dokuz Eylul University, diagnosed with migraine (International Classification of Diseases, 10th Revision (ICD-10) code G43 and its subcategories), and experiencing migraine attacks less than once every three months.

The sample size was calculated considering a 5% margin of error, 80% power, medium effect size, and a 15% dropout rate, resulting in a required sample size of 86 participants. These participants will be equally divided into control and intervention groups. The sample size was calculated using G*POWER 3.1.9.2.

### Inclusion and exclusion criteria

Participants aged 18–50 years, owning an internet-enabled communication device, and willing to participate were included. Eligible participants were required to have a migraine diagnosis with attack frequency of less than three months and meet specific migraine diagnostic criteria. Exclusion criteria included severe anatomical abnormalities of the airway, pregnancy, diagnosed psychiatric disorders, current use of psychiatric medications, and significant speech or hearing problems. Additionally, patients with chronic comorbidities such as diabetes, hypertension, Chronic Obstructive Pulmonary Disease (COPD), asthma, and cardiac diseases were excluded. Only individuals without active complaints, no history of related medication use, and clean records in electronic health systems were considered for inclusion. None of the participants were on prophylactic treatment before the study, and any participants who would have started prophylactic treatment during the study were to be excluded, although this did not occur. Every participant continued their usual treatment, and no interventions were made to alter their pharmacological therapy.

### Participant recruitment and randomization

Data were collected through face-to-face interviews conducted between 01.1.2023 and 30.1.2023 at the FHCs of Dokuz Eylul University. Initially, eligible participants from each six different FHCs were listed separately and subsequently selected using a random number generator. The selection conducted to achieve an equal number of participants within each center. These centers were then cluster-randomized into two groups: three were assigned to the intervention group and three to the control group. This cluster randomization process was also performed using a random number generator, and the entire procedure was carried out by the researcher (Figure 1).

### Data collection tools

#### Personal data form

A questionnaire was developed to capture sociodemographic information such as gender, marital status, educational level, employment status, income status, and age.

#### Migraine Disability Assessment Scale (MIDAS)

Migraine Disability Assessment Scale (MIDAS) assesses the level of disability in migraine patients by evaluating daily life activities affected by migraine attacks. The MIDAS consists of five questions that assess the number of days migraines have impacted various aspects of daily life over the past three months. These include days missed at work or school, days of reduced productivity at work or school, days missed doing household work, days of reduced productivity in household work, and days missed in social or leisure activities. Each question is scored based on the number of days affected, with higher scores indicating greater disability. Additionally, the MIDAS includes questions on headache frequency (number of migraine days) and severity (measured using a Visual Analog Scale, VAS) to provide a comprehensive assessment of the migraine’s impact on the individual’s life. The scale’s Turkish validity and reliability were confirmed by Ertaş et al. in 2004, with a Cronbach’s alpha of 0.76.

### Pre-randomization and baseline evaluation

All patients attended an individual session at their respective FHCs, where they received standardized education on migraine management, including topics such as migraine triggers, available treatment options, and recommended lifestyle modifications. This initial education ensured a uniform baseline level of knowledge across participants. Following this, an initial evaluation was conducted individually with both the intervention and control groups to establish a baseline for the study. Participants from both groups completed the required forms in the presence of the researcher, who provided assistance as needed to minimize misunderstandings and potential errors. This approach ensured accuracy and consistency in data collection across both groups.

### Intervention

Patients in the intervention group participated in a 30-min, one-on-one session with the researcher at their respective FHCs. During this session, the researcher provided individualized instruction and practice in the ANB technique. The researcher demonstrated the technique and guided each participant through the exercises, ensuring correct understanding and performance. To support ongoing practice and adherence, participants in the intervention group received comprehensive written materials, including step-by-step instructions and additional guidance on the ANB technique (Supplementary Material 1).

### Follow-up and outcome

Throughout the study, participants in the intervention group received weekly phone calls to support adherence to the ANB technique. Each weekly call, lasting approximately 5–10 min, focused on the following topics:

Adherence to the exercise regimen and frequency of practice

Correct technique for performing ANB

Addressing any questions or concerns related to the exercise

Encouragement and motivation to continue the practice

Additionally, both the intervention and control groups received monthly phone calls, each lasting 10–20 min, which provided general support and follow-up related to their migraine conditions. Topics covered in these monthly calls included:

Monitoring any changes in migraine frequency, duration, and intensity

General health updates and well-being checks

Guidance on lifestyle or dietary adjustments that may help manage migraines

Emotional and psychological support related to migraine management.

At the end of the three-month period, participants from both groups were contacted by phone, and the MIDAS questionnaire was administered again. The primary outcomes were the changes in headache frequency and severity, as recorded by participants in a diary. Headache frequency was defined as the number of migraine days. Severity was measured using the VAS from 0 to 10. Participants were provided with instructions on how to identify and record migraine-specific headaches. The secondary outcomes focused on changes in the MIDAS score. A migraine day was defined as any day with migraine symptoms. Attacks lasting over 24 h were considered one attack. At the conclusion of the study, the control group was informed about the exercise technique and invited to a session to learn and practice it. There were no complications reported during the study.

### Statistical analysis

Dependent variables included pain intensity, pain frequency, and MIDAS score. Independent variables included sociodemographic characteristics and the use of breathing techniques. Data analysis was performed using IBM SPSS Statistics V27.0. Descriptive analyses were conducted for all variables. For comparisons, means and standard deviations were used for continuous variables, and frequencies and percentages for categorical variables. Chi-square tests were used for categorical independent and dependent variables. To test the distributions, the Kolmogorov–Smirnov test was used. The MIDAS scores, which followed a normal distribution, were analyzed using independent t-tests for comparisons between the intervention and control groups and paired t-tests for within-group comparisons. For attack frequency and attack severity, which did not follow a normal distribution, the Mann–Whitney U test was used for between-group comparisons, and the Wilcoxon test was employed for within-group comparisons. Analyses followed the intention-to-treat principle using the multiple imputation method, with a significance level set at p < 0.05.

## Results

### Baseline findings

Eighty-six migraine patients aged 18–50 years participated in the study. The mean age of the participants was 39.11 ± 7.21 years, and 51.16% (44 participants) were aged 40–50 years. Among the participants, 75.58% (65 individuals) were female, 80.23% (69 participants) were married, and 40.70% (35 participants) had university-level or higher education, 93.02% (80 participants) were employed, and 33.72% (29 participants) had income less than expenses. There was no statistically difference between the control and intervention groups (Table 1).

**Table 1.** Sociodemographic characteristics of participants

Sociodemographic characteristics	Intervention n (%)	Control n (%)	Total n (%)	*P*-value
**Gender**				0.45
Female	34 (39.53)	31 (36.05)	65 (75.58)	
Male	9 (10.47)	12 (13.95)	21 (24.42)	
**Marital status**				0.37
Married	35 (40.70)	34 (39.53)	69 (80.23)	
Single	6 (6.98)	4 (4.65)	8 (9.30)	
Divorced/widowed	2 (2.33)	5 (5.82)	7 (8.14)	
**Educational level**				0.45
Primary school	16 (18.60)	13 (15.12)	18 (20.93)	
High school	11 (12.79)	11 (12.79)	22 (25.58)	
University or higher	16 (18.60)	19 (22.09)	35 (40.70)	
**Employment status**				0.40
Employed	39 (45.35)	41 (47.67)	80 (93.02)	
Unemployed/retired	4 (4.65)	2 (2.33)	6 (6.98)	
**Income status**				0.78
Income equals expenses	14 (16.28)	11 (12.79)	25 (29.07)	
Income less than expenses	15 (17.44)	14 (16.28)	29 (33.72)	
Income more than expenses	14 (16.28)	18 (20.93)	32 (37.21)	
**Age (years)**				0.32
18–24	4 (4.65)	4 (4.65)	8 (9.30)	
25–39	16 (18.60)	18 (20.93)	34 (39.53)	
40–50	23 (26.74)	21 (24.42)	44 (51.16)	

### Attack frequency, severity, and MIDAS score

While the frequency and severity of attacks did not follow a normal distribution, the MIDAS score was normally distributed. The median attack frequency was lesser among the intervention groups after the intervention (*P* = 0.002), while no statistically significant difference was existed before (*P* = 0.415). There was no statistically significant difference between control and intervention groups neither before nor after the intervention (*P*-value was equal to 0.252 and 0.074 orderly) (Table 2).

**Table 2.** Comparison of attack frequencies and severities between groups before and after the ıntervention

	Intervention (Median)	Control (Median)	*P*-value
**Before interventıon**			
Attack frequency	15	18	0.416
Attack severity	5	8	0.252
**After interventıon**			
Attack frequency	12	17	0.002
Attack severity	4	7	0.074

Also MIDAS score was lesser within the intervention group after the intervention (p = 0.003), though there was no statistically significant difference between the groups before (*P* = 0.484) (Table 3).

**Table 3.** Comparison of MIDAS scores before and after the ıntervention

	Intervention (M ± SD)	Control (M ± SD)	*P*-value	Cohen’s d
**Before intervention**				
MIDAS score	33.06 ± 10.12	34.67 ± 11.06	0.485	10.60
**After intervention**				
MIDAS score	27.34 ± 9.27	33.96 ± 10.76	0.003	10.04

### Within-group comparisons

Attack frequency, severity and MIDAS score was significantly lower in the intervention group (*P* = 0.001, *P* = 0,001, and *P* = 0,001 orderly) on where as there was no significant change in the control group for attack frequency and MIDAS score before and after intervention (*P* = 0.589 and *P* = 0,079 orderly). Only attack severity was decreased within the control group after intervention (*P* = 0.001) (Table 4, 5).

**Table 4.** Examination of changes in attack frequencies and severities within groups between the beginning and end

Groups	Attack frequency			Attack severity		
	Before Median (Min-Max)	After Median (Min-Max)	*P*-value	Before Median (Min-Max)	After Median (Min-Max)	*P*-value
**Intervention**	15 (4–31)	12 (4–29)	0.001	5 (5–10)	4 (4–9)	0.001
**Control**	18 (5–33)	17 (3–35)	0.589	8 (5–10)	7 (4–9)	0.001

**Table 5.** Examination of changes in MIDAS scores within groups before and after the ıntervention

Groups	MIDAS score (M ± SD) before intervention	MIDAS score (M ± SD) after intervention	*P*-value
**Intervention**	33.06 ± 10.12	27.34 ± 9.27	0.001
**Control**	34.67 ± 11.06	33.96 ± 10.76	0.079

## Discussion

Our study highlights the importance of using the ANB technique as a non-pharmacological method for managing migraine headaches and its impact on patients’ migraine-related disability. As a non-invasive, non-pharmacological approach, the breathing technique can be easily applied without side effects or additional costs for both practitioners and patients. This technique can be integrated into patients’ daily routines and may have positive effects on migraine control with regular practice.

### Relevance of the study group

Migraine prevalence tends to peak around the age of 40 years in both men and women and decreases with age, as supported by studies showing similar age distribution patterns (Piccinini *et al.*, 2023). Also it is much prevalent among women (Özdemir *et al.*, 2014). The average age of participants in our study was 39.11 ± 7.21 years with the number of female participants being three times greater than that of male participants.

According to our study results, the participants’ baseline MIDAS score was 33.87 ± 1.14, with an attack frequency of 17.50 ± 0.68 (over a three-month period) and an attack severity of 8.08 ± 0.17. Medrano Martínez *et al.* (2021) reported a mean MIDAS score of 39.88 and an average attack severity of 7.49, while Guilbot *et al.* (2017) found a monthly attack frequency of 4.9. On the other hand, the mean MIDAS score in our study was comparable to national data from Turkey, where the average MIDAS score is 35.32 ± 36.59 (Çimen, 2019). These findings affirm the relevance of our study population, as their characteristics reflect both national and international migraine data. Therefore, while our results are applicable to our study population, caution should be exercised when considering the generalizability of our findings to broader populations, as RCTs are inherently limited to the populations in which they are conducted.

Many studies have reported higher MIDAS scores in patients with comorbid conditions, such as depression (43.0 ± 43.8) (Hans *et al.*, 2023). Also Sengul *et al.* (2014) state that higher MIDAS scores correlate with anxiety and sleep disorders. This observation suggests that patients with higher baseline MIDAS scores, potentially associated with comorbid conditions, might respond more favorably to ANB, as ANB has shown improvements in various health conditions and comorbidities (Jahan *et al.*, 2021). However, further studies are needed to substantiate these findings and explore the extent of ANB’s effectiveness in this context.

Importantly, there were no significant differences between the intervention and control groups in terms of sociodemographic variables, MIDAS scores, attack frequency, or attack severity at baseline. This balance between the groups shows our study’s randomization process, ensuring that the comparison of outcomes post-intervention is methodologically sound.

### Outcomes

For our intervention’s effectiveness, it is essential to note that there were no significant differences between the two groups in terms of sociodemographic variables, MIDAS score, attack frequency, and attack severity prior to the intervention. Therefore, no adjustment analysis was necessary for the results obtained.

The intervention group showed a significant reduction in attack frequency post-intervention, while no significant change was observed in the control group. This suggests that ANB effectively reduces the frequency of migraine attacks. The intervention group also exhibited a significant reduction in MIDAS scores, indicating improved daily functioning and migraine-related disability. The control group showed no significant change, underscoring the effectiveness of the breathing technique. But in contrary, there was no significant difference on attack severity between the groups, suggesting that while the intervention may reduce severity, other factors also contribute to this outcome. These findings suggest that the intervention effectively reduces attack frequency and improves MIDAS scores. However, its effect on attack severity was not as pronounced, which may be due to several factors. The intervention may influence the pathways that initiate migraine attacks but not the mechanisms affecting pain intensity. Further research is needed to explore the intervention’s impact on these pathways.

Hypoxia is known to trigger migraine-like and aura attacks by causing arterial dilation (Arngrim *et al.*, 2016). ANB may reduce this hypoxic state, potentially decreasing the frequency of migraine attacks. However, it does not seem to affect cortical spreading depression (CSD), the electrophysiological event underlying migraine auras (Kudo *et al.*, 2008). This could explain the lack of significant effect on attack severity.

The duration of the intervention may also have been insufficient to affect attack severity. Future studies should consider longer intervention periods to assess sustained effects. Previous studies on alternative migraine treatments have typically involved three-month periods (Fuglsang *et al.*, 2018; Xie *et al*., 2022).

Another consideration is that participants may not have performed the ANB technique correctly during an attack, affecting its efficacy on attack severity. Detailed examination of the timing and application of the intervention in future research is necessary.

Both groups showed improvements in attack severity from baseline to post-intervention, which could be attributed to all participants receiving the latest migraine information, while continuing their usual treatment, thereby increasing disease management awareness and resulting in similar improvement potentials in both groups.

These results indicate that patient awareness of migraine symptoms is crucial for effective management. Recognizing trigger factors and taking appropriate measures are vital. Additionally, regular monitoring and appropriate treatment recommendations by healthcare professionals play a critical role in managing migraine symptoms (Rosignoli *et al.*, 2022).

This study is the first to investigate the use of ANB in migraine patients, making a significant contribution to the literature.

### Limitations and strengths of the study

This study was conducted in a primary care setting, providing insights into how migraine treatment can be implemented within basic health services, which is important for broader patient access. Including participants without comorbidities allows for a more direct interpretation of results concerning migraine itself, minimizing the influence of other health conditions. As one of the first studies to examine the effects of ANB in migraine treatment, it contributes new knowledge to the field. Using cluster sampling reduced the risk of contamination between groups, enhancing the reliability of the results.

The intervention period may have been too short to evaluate long-term effects, necessitating longer follow-up in future studies. The long-term impact of the intervention on behavioral and symptomatic outcomes was not assessed, leaving a gap in understanding its lasting effects. Increased contact between the researcher and the intervention group could have introduced observer bias; however, this was mitigated by providing both groups with equal information and using cluster sampling to randomize intervention and control groups. Also tracking changes in drug usage frequency could provide important insights into this technique’s potential as an alternative or complementary migraine treatment. Future studies will incorporate this measure to enhance the comprehensiveness of the findings. Despite conducting multiple training sessions and follow-up meetings to ensure participants executed the breathing techniques correctly, there remains the possibility that some participants did not practice the techniques accurately. This potential inconsistency could influence the integrity of the results and does not entirely eliminate the influence of placebo effects or participant variability. To address these concerns, we recommend that future studies incorporate a comparison with a similar but inactive intervention. The study’s reliance on measurable evaluation instruments might have limited the inclusion of biological or objective data, which could enhance the reliability of the results. Lastly, the study’s design may have led to more frequent contact between the researcher and the intervention group compared to the control group, potentially introducing observer bias.

## Conclusion

This study suggests that ANB may be beneficial in reducing the frequency of migraine attacks and improving migraine-related disability. Future research should confirm the effectiveness of this technique and explore its broader applications. A multidisciplinary approach that includes primary care, with regular monitoring and comprehensive treatment recommendations by healthcare professionals, is essential. Further studies are needed to validate the efficacy of ANB and promote its use among a wider patient population.

## Supporting information

Çöme et al. supplementary material Çöme et al. supplementary material

## Supplementary material

The supplementary material for this article can be found at https://doi.org/10.1017/S1463423625000064

## References

[ref2] Amiri P , Kazeminasab S , Nejadghaderi SA , Mohammadinasab R , Pourfathi H , Araj-Khodaei M , Sullman MJM , Kolahi A-A and Safiri S (2022) Migraine: a review on its history, global epidemiology, risk factors, and comorbidities. Frontiers in Neurology 12, 800605. 10.3389/fneur.2021.800605 35281991 PMC8904749

[ref3] Arngrim N , Schytz HW , Britze J , Amin FM , Vestergaard M. B. , Hougaard A , Wolfram F , de Koning PJH , Olsen KS , Secher NH. , Larsson HBW , Olesen J and Ashina M (2016) Migraine induced by hypoxia: an MRI spectroscopy and angiography study. Brain: A Journal of Neurology 139(Pt 3), 723–737. 10.1093/brain/awv359 26674653

[ref6] Ciarambino T , Sansone G , Menna G , Para O , Signoriello G , Leoncini L and Giordano M (2021) Oxygen therapy in headache disorders: a systematic review. Brain Sciences 11(3), 379. 10.3390/brainsci11030379 33802647 PMC8002555

[ref7] Çimen A. et al.,. (2019) Çimen Atalar A, Yalın OÖ, Aslan H, Baykan B. Ailede migren öyküsü bulunmasının migren özelliklerine etkisi nedir? [What is the impact of having a family history of migraine on migraine characteristics?]. Agri. 2019 Jul;31(3):113-121. Turkish. Doi: 10.14744/agri.2019.26042. PMID: 31736021. 31736021

[ref9] Fuglsang CH , Johansen T , Kaila K , Kasch H and Bach FW (2018) Treatment of acute migraine by a partial rebreathing device: a randomized controlled pilot study. Cephalalgia: An International Journal of Headache 38(10), 1632–1643. 10.1177/0333102418797285 30134739 PMC6158684

[ref10] Gawde P , Shah H , Patel H , Bharathi KS , Patel N , Sethi Y and Kaka N (2023) Revisiting migraine: the evolving pathophysiology and the expanding management armamentarium. Cureus 15(2), e34553. 10.7759/cureus.34553 36879707 PMC9985459

[ref11] Guilbot A , Bangratz M , Ait Abdellah S and Lucas C (2017) A combination of coenzyme Q10, feverfew and magnesium for migraine prophylaxis: a prospective observational study. BMC Complementary and Alternative Medicine 17(1), 433. 10.1186/s12906-017-1933-7 28854909 PMC5577764

[ref12] Gupta J and Gaurkar SS (2022) Migraine: an underestimated neurological condition affecting billions. Cureus 14(8). 10.7759/cureus.28347 PMC9506374 36168353

[ref13] Hans A , Stonnington CM , Zhang N , Butterfield R and Friedman DI (2023) The impact of resilience on headache disability as measured by the migraine disability assessment (MIDAS). Headache 63(6), 743–750. 10.1111/head.14518 37218745

[ref14] Jahan I , Begum M , Akhter S , Islam MZ , Jahan N , Samad N , Das P , Rahman NA and Haque M (2021) Effects of alternate nostril breathing exercise on cardiorespiratory functions in healthy young adults. Annals of African Medicine 20(2), 69–77. 10.4103/aam.aam_114_20 34213471 PMC8378456

[ref15] Kudo C , Nozari A , Moskowitz MA and Ayata C (2008) The impact of anesthetics and hyperoxia on cortical spreading depression. Experimental Neurology 212(1), 201–206. 10.1016/j.expneurol.2008.03.026 18501348 PMC2459317

[ref16] Medrano Martínez V , Francés Pont I , Hernández Rubio L , González Fernández L , Fernández Izquierdo S and Mallada Frechin J (2021) Perception of the validity of the migraine disability assessment questionnaire in a population of patients with chronic migraine. Neurologia 36(9), 692–697. 10.1016/j.nrleng.2020.08.004 34752347

[ref18] Nivethitha L , Mooventhan A and Manjunath NK (2016) Effects of various Prānāyāma on cardiovascular and autonomic variables. Ancient Science of Life 36(2), 72. 10.4103/asl.ASL_178_16 28446827 PMC5382821

[ref19] Ozdemir G , Aygül R , Demir R , Ozel L , Ertekin A and Ulvi H (2014) Migraine prevalence, disability, and sociodemographic properties in the eastern region of Turkey: a population-based door-to-door survey. Turkish Journal of Medical Sciences 44(4), 624–629. 25551933

[ref20] Piccininni M , Brinks R , Rohmann JL and Kurth T (2023) Estimation of migraine prevalence considering active and inactive states across different age groups. The Journal of Headache and Pain 24(1), 83. 10.1186/s10194-023-01624-y 37430201 PMC10334692

[ref21] Puledda F , Tassorelli C and Diener HC (2023) New migraine drugs. Cephalalgia 43(3), 03331024221144784. 10.1177/03331024221144784 36786364

[ref22] Rosignoli C , Ornello R , Onofri A , Caponnetto V , Grazzi L , Raggi A , Leonardi M and Sacco S (2022) Applying a biopsychosocial model to migraine: rationale and clinical implications. The Journal of Headache and Pain 23(1), 100. 10.1186/s10194-022-01471-3 35953769 PMC9367111

[ref23] Russo MA , Santarelli DM and O’Rourke D (2017) The physiological effects of slow breathing in the healthy human. Breathe 13(4), 298–309. 10.1183/20734735.009817 29209423 PMC5709795

[ref24] Santaella DF , Devesa CRS , Rojo MR , Amato MBP , Drager LF , Casali KR , Montano N and Lorenzi-Filho G (2011) Yoga respiratory training improves respiratory function and cardiac sympathovagal balance in elderly subjects: a randomised controlled trial. BMJ Open 1(1), e000085. 10.1136/bmjopen-2011-000085 PMC3191432 22021757

[ref25] Schindler EAD , Wright DA , Weil MJ , Gottschalk CH , Pittman BP and Sico JJ (2018) Survey analysis of the use, effectiveness, and patient-reported tolerability of inhaled oxygen compared with injectable sumatriptan for the acute treatment of cluster headache. Headache: The Journal of Head and Face Pain 58(10), 1568–1578. 10.1111/head.13405 30221765

[ref26] Sengul Y , Sengul HS , Bakim B , Yucekaya SK , Yucel S and Akgun M (2014) Sleep disturbances and excessive daytime sleepiness in migraine: A comparison between comorbidities and disability: Sleep disturbances in migraine. Sleep and Biological Rhythms 13(1), 76–84. 10.1111/sbr.12087

[ref27] Singhal AB , Maas MB , Goldstein JN , Mills BB , Chen DW , Ayata C , Kacmarek RM and Topcuoglu MA (2017) High-flow oxygen therapy for treatment of acute migraine: a randomized crossover trial. Cephalalgia: An International Journal of Headache 37(8), 730–736. 10.1177/0333102416651453 27206964

[ref28] Wang M , Lan D , Dandu C , Ding Y , Ji X and Meng R (2023) Normobaric oxygen may attenuate the headache in patients with patent foramen povale and migraine. BMC Neurology 23(1), 44. 10.1186/s12883-023-03059-z 36707824 PMC9881355

[ref29] Wilson JR , Foresman BH , Gamber RG and Wright T (1998) Hyperbaric oxygen in the treatment of migraine with aura. Headache 38(2), 112–115. 10.1046/j.1526-4610.1998.3802112.x 9529766

[ref31] Xie YJ , Tian L , Hui SSC , Qin J , Gao Y , Zhang D , Ma T , Suen LKP , Wang, HH , Liu ZM , Hao C , Yang L and Loke Yuen A (2022) Efficacy and feasibility of a 12-week Tai Chi training for the prophylaxis of episodic migraine in Hong Kong Chinese women: A randomized controlled trial. Frontiers in Public Health 10, 1000594. 10.3389/fpubh.2022.1000594 36582390 PMC9792997

