# Supplementary material for: The impact of alternate nostril breathing on the severity and frequency of migraine attacks: a randomized control trial
Source: Prim Health Care Res Dev. 2025 Feb 14;26:e12. doi: 10.1017/S1463423625000064 (PMC11836872; doi:10.1017/S1463423625000064)
Supplement: Çöme et al. supplementary material [file S1463423625000064sup001.pdf]

Application of Alternate Nostril Breathing Technique

To begin, sit comfortably on a chair or cushion with your spine straight. If leaning against a chair or sofa causes your back to recline, place a cushion behind your lower back for additional support. Alternatively, you may sit cross-legged on the floor or on a meditation cushion. Closing your eyes during the practice can help you focus inward; however, if this feels uncomfortable, you may fix your gaze on a single point on the wall or floor. It is recommended to clear your nasal passages before starting the practice to facilitate smoother breathing. If you experience any discomfort during the session, feel free to stop at any time.

During this practice, you will use only one nostril at a time for each breath cycle, alternating nostrils with the help of your fingers. To close a nostril, gently press on the cartilage at the base where it meets the nasal bone. Avoid applying excessive pressure to prevent unintentionally constricting the open nostril. If you are using your right hand, use your thumb to close the right nostril and your ring finger (fourth finger) to close the left nostril. You can rest your index and middle fingers on your forehead for added support, or keep them relaxed or folded into your palm if that feels more natural. For left-hand use, simply reverse the fingers' roles. If one hand becomes fatigued, switch to the other.

You may notice differences in airflow between the two nostrils, which is normal. If one nostril feels obstructed, use your free hand to gently pull the skin of your cheek outward to open the nasal passage and facilitate easier breathing.

Start by taking a deep breath through both nostrils. Exhale and close your right nostril with your thumb, breathing out through your left nostril. Then, inhale through the left nostril. Close the left nostril with your ring finger as you exhale through the right nostril. Next, inhale through the right nostril to complete one full cycle. Continue in this pattern: exhale and inhale through the same nostril before switching to the other.

To simplify, remember that each cycle involves exhaling and inhaling from one nostril before switching sides. Adjust the duration of the practice to suit your comfort level. You may also explore extending the exhalation phase to be longer than the inhalation phase, as longer exhalations can promote relaxation and a sense of calm. Ensure this extension does not create tension or stress.

The practice should begin and end with an exhalation through the left nostril.

As part of the exercise protocol, it is expected that you practice this breathing exercise at least three sessions per day. Each session should include nine complete cycles.

For any questions or issues you may wish to discuss during weekly evaluations, you are welcome to reach out through regular phone calls, video calls, or messaging platforms to clarify any uncertainties and receive guidance on your practice to ensure that you are following the technique correctly and maximizing its benefits.
